# Additional risk factors improve mortality prediction for patients hospitalized with influenza pneumonia: a retrospective, single-center case–control study

**DOI:** 10.1186/s12890-022-02283-6

**Published:** 2023-01-16

**Authors:** Yu Bai, Yiqun Guo, Li Gu

**Affiliations:** Department of Infectious Diseases and Clinical Microbiology, Beijing Institute of Respiratory Medicine and Beijing Chao-Yang Hospital, Capital Medical University, No.8 Worker’s Stadium South Road, Chaoyang District, Beijing, China

**Keywords:** Influenza, Pneumonia, D-dimer, Ferritin, Risk factor

## Abstract

**Background:**

Severe influenza, especially influenza pneumonia, causes large numbers of deaths each year. Some patients who develop severe influenza have no known risk factors. In this study we investigated risk factors for mortality of patients with influenza A-related pneumonia who have different basic conditions. We also evaluated the power of pneumonia severity assessment tools in Chinese patients hospitalized with influenza A-related pneumonia. Together, these results could provide a basis for a screening method that has improved ability for the early identification of critical patients who will have poor prognoses in clinical practice.

**Methods:**

This single-center, retrospective case–control study included 152 adult patients with severe influenza over six influenza seasons. Data for diagnoses and demographics, as well clinical data, laboratory findings, treatment methods, 30-day and 60-day outcomes of the patients were collected. Patients who had any of the risk factors for severe influenza were included in the high-risk group, and those that had no known risk factors were included in the low-risk group.

**Results:**

The PSI, CURB-65 and PIRO-CAP tools all underestimated the mortality rate of patients hospitalized with influenza A-related pneumonia, and this underestimate was more pronounced for low-risk patients. D-dimer (Odds ratio [OR] = 1.052, 95% confidence interval [CI] 1.001–1.106, *p* = 0.045) and direct bilirubin (OR = 1.143, 95%CI 1.049–1.246, *p* = 0.002) were independent risk factors for mortality of patients with influenza A-related pneumonia. When used in combination with ferritin and D-dimer, the area under receiver operator characteristic curve (AUC_ROC_) was 0.851 (95%CI 0.780–0.922, *p* < 0.001), 0.840 (95%CI 0.763–0.916, *p* < 0.001) and 0.829 (95%CI 0.748–0.911, *p* < 0.001) for PSI, CURB-65 and PIRO-CAP, respectively, which was higher than that obtained using PSI, CURB-65 and PIRO-CAP alone.

**Conclusions:**

The findings demonstrate that currently used community-acquired pneumonia (CAP) scoring systems could underestimate the risk of influenza A-related pneumonia mortality. D-dimer was shown to be an independent risk factor of mortality for influenza A-related pneumonia in hospitalized patients, and a combination of D-dimer with ferritin could improve the predictive value of PSI, CURB-65 and PIRO-CAP for adverse prognoses of patients with influenza A-related pneumonia.

**Supplementary Information:**

The online version contains supplementary material available at 10.1186/s12890-022-02283-6.

## Introduction

Infection with influenza virus can cause diseases related to lower respiratory infection, including community-acquired pneumonia (CAP), acute exacerbation of chronic obstructive pulmonary disease (AECOPD), and acute bronchitis [1]. Influenza virus is a major pathogen of CAP [2] and is the cause of numerous deaths. Influenza virus infections increase the burden on both global healthcare systems and economies. Prior to the COVID-19 pandemic, the incidence of influenza-related lower respiratory infection was 151.8/100,000, and the mortality rate was 0.8/100,000, according to predictions based on relevant data [3]. Meanwhile, a population-based study in China established a regression model based on 5-year data for patients in 22 provinces that estimated that 88,100 extra influenza-related deaths occurred annually in China, accounting for 8.2% of all deaths caused by respiratory diseases [4]. Several important studies have identified high-risk groups and risk factors for severe influenza. The elderly and children are at increased risk for severe influenza and advanced age, pregnancy, underlying diseases (excluding hypertension) and obesity are also known risk factors for severe influenza [5]. Moreover, when combined with respiratory bacterial and fungal infections, bacteremia [6] and early glucocorticoid use [7] have also been shown to be risk factors for acute respiratory distress syndrome (ARDS) or death.

In clinical practice, existing risk factors may not accurately depict vulnerable populations for severe influenza, and a considerable proportion of low-risk populations progresses to critical illness or even death after influenza virus infection, which presents additional challenges for the diagnosis and treatment of influenza pneumonia. Due to the high incidence and mortality rate of influenza-related lower respiratory infections, identifying patients with influenza infection who may progress to severe disease in the early stages is important for determining the most effective treatments for clinical practices. However, the clinical characteristics of influenza-related pneumonia are distinct from CAP induced by other pathogens. All currently used assessment tools for disease conditions associated with pneumonia, such as CURB-65, pneumonia severity index (PSI), and PIRO-CAP, underestimated mortality and failed to predict intensive care unit (ICU) admission [8]. Other studies have also confirmed that disease assessment scores, including those generated by the abovementioned tools, are not good predictors of ICU admission, mechanical ventilation, or death in patients hospitalized with influenza pneumonia [9, 10].

Therefore, this study aimed to investigate risk factors for severe influenza A mortality including patients in low-risk populations in a hospital-based case–control study. We evaluated the 30 and 60-day death prediction of previous pneumonia severity using evaluation tools for Chinese patients hospitalized with severe influenza A, and preliminarily explored how existing disease assessment tools could be improved to provide a more meaningful screening method for early clinical identification of patients with severe influenza A.

## Methods

### Study population and design

This study was a single-center, retrospective case–control study that included adult patients with severe influenza who were hospitalized in the Department of Infectious Diseases and Clinical Microbiology and Department of Respiratory and Critical Care Medicine at Beijing Chao-Yang Hospital of the Capital Medical University across six influenza seasons: 2013–2014 to 2018–2019. Inclusion criteria included: (1) age ≥ 18 years-old and (2) confirmed positive for influenza A virus by laboratory test. In addition, patients had to meet the following criteria for severe influenza by exhibiting one of the following characteristics[5]: (1) persistent hyperpyrexia for > 3 d, accompanied by cough, purulent sputum, or chest pain; (2) high respiration rate, dyspnea, or cyanosis; (3) changes in consciousness, such as lags in response, somnolence, agitation, and seizure; (4) severe vomiting and diarrhea, manifested by dehydration; (5) accompanying pneumonia; and (6) worsening of underlying diseases.

Exclusion criteria were: (1) influenza A patients who did not meet the above definitions of severe influenza; (2) cases for which the patient's condition was difficult to evaluate due to incomplete clinical data; (3) patients with unknown prognosis and no prognostic information that could be obtained through telephone follow-up.

### Data collection

The diagnosis, demographic data, clinical data, laboratory findings, treatments, and 30-day and 60-day outcomes for the patients were collected from in-hospital medical records and the case collection system. In order to be able to compare with data from previous studies, 30-day outcome was used in the evaluation of the disease assessment tools. But in this study 23 patients were still hospitalized at day 30, and all patients were discharged or died before 60th day. Therefore, it seems that the application of 60-day mortality is more realistic. The study was conducted in accordance with the ethical regulations of the Institutional Review Board (IRB) of Beijing Chao-Yang Hospital and the World Medical Association Declaration of Helsinki. The protocol was approved by the IRB of Beijing Chao-Yang Hospital (2015-KE-158).

Medical attendance and demographic data included age, sex, time from disease onset to admission, length of stay, underlying diseases, and body-mass index (BMI). Clinical data included use of mechanical ventilation, use of vasoactive drugs, use of continuous renal replacement therapy (CRRT), use of anti-influenza drugs, and ICU admission. Laboratory tests included routine blood tests, liver and kidney function, blood lipids, albumin, coagulation function, inflammatory indicators, T cell subsets, sputum bacteria and mycological results, and blood culture results. PSI, CURB-65 and PIRO-CAP scores at admission were calculated from relevant admission examination results. All patients were observed during hospitalization, and the endpoint was day 60 of hospitalization or death. We also observed and recorded 30-day deaths for comparison with other assessment tools. For patients discharged within 60 days, survival was determined by outpatient or telephone follow-up on day 60 after the date of admission.

Patients were classified into a low-risk group and high-risk group according to the absence of risk factors of severe influenza, and relevant studies were then performed. Patients who had any of the risk factors for severe influenza were included in the high-risk group, and those who had no known risk factors were included in the low-risk group.

### Definitions

Influenza infection was diagnosed by a positive result on antigen test, or nucleic reverse transcriptase polymerase chain reaction (RT-PCR) performed on nasopharyngeal or throat swabs, sputum or bronchoalveolar lavage.

Severe pneumonia was diagnosed according to guideline of the American thoracic society and Infectious Diseases Society of America [11]: Validated definition includes either one major criterion or three or more minor criteria. Major criteria: (1) Septic shock with need for vasopressors; (2) Respiratory failure requiring mechanical ventilation. Minor criteria: (1) Respiratory rate ≥ 30 breaths/min; (2) PaO_2_/FiO_2_ ratio ≤ 250 mmHg; (3) Multilobar infiltrates; (4) Confusion/disorientation; (5) Uremia (blood urea nitrogen level ≥ 20 mg/dl); (6) Leukopenia (white blood cell count < 4,000 cells/μl); (7) Thrombocytopenia (platelet count < 100,000/μl); (8) Hypothermia (core temperature < 36 °C); (9) Hypotension requiring aggressive fluid resuscitation.

ARDS was diagnosed according to the Berlin definition: acute onset within 1 week, bilateral lung opacities, no evidence of cardiac failure-related hydrostatic edema by echocardiography, and PaO_2_/FiO_2_ < 300 mmHg with positive end-expiratory pressure ≥ 5 cm H_2_O [12].

The risk factors for severe influenza were defined as: (1) elderly, i.e., aged ≥ 65 years-old; (2) accompanied by certain diseases or conditions, such as chronic respiratory diseases, cardiovascular diseases (except for hypertension), renal diseases, liver diseases, hematological diseases, neurological diseases, neuromuscular diseases, metabolic disorders, endocrine diseases, and immunosuppression (including use of immunosuppressants, or immunocompromised status due to HIV infection); (3) obesity [BMI > 30. BMI = body weight (kg)/height (m)^2^]; or 4) pregnant or perinatal women [5].

### Statistical analysis

Statistical analyses were performed using SPSS Statistics V.23 (IBM Inc., Chicago, Illinois, USA) and MedCalc V.19.6.4 (MedCalc Software, Ostend, Belgium). Data were presented as either the median with interquartile range (IQR) or mean ± standard deviation (SD) for numerical variables, count and percentage for categorical variables where appropriate. Continuous variables with normal distribution were compared using t-tests, while those with non-normal distribution were compared using Mann–Whitney U tests. Categorical variables were compared using Chi-Square Tests. Variables that differed significantly between survival and mortality groups were considered as potential risk factors. Logistic regression analysis was performed to screen the risk factors for influenza A-related pneumonia 60-day mortality of patients. The results were presented as estimates of relative risk expressed as a odds ratio (OR) with a 95% confidence interval (CI). Statistical significance was established at *p* < 0.05. Receiver operator characteristic (ROC) curves were plotted to calculate the area under curve (AUC) for different disease prediction tools, and the logistic regression model for combination diagnoses was established based on the findings. The prediction probabilities of the combinations were calculated, the ROC curve was plotted according to the predictive probability, and then the AUC for different combination diagnoses was calculated.

## Results

### Demographic characteristics, underlying diseases, and prognoses of patients with severe influenza A

**Fig. 1:**
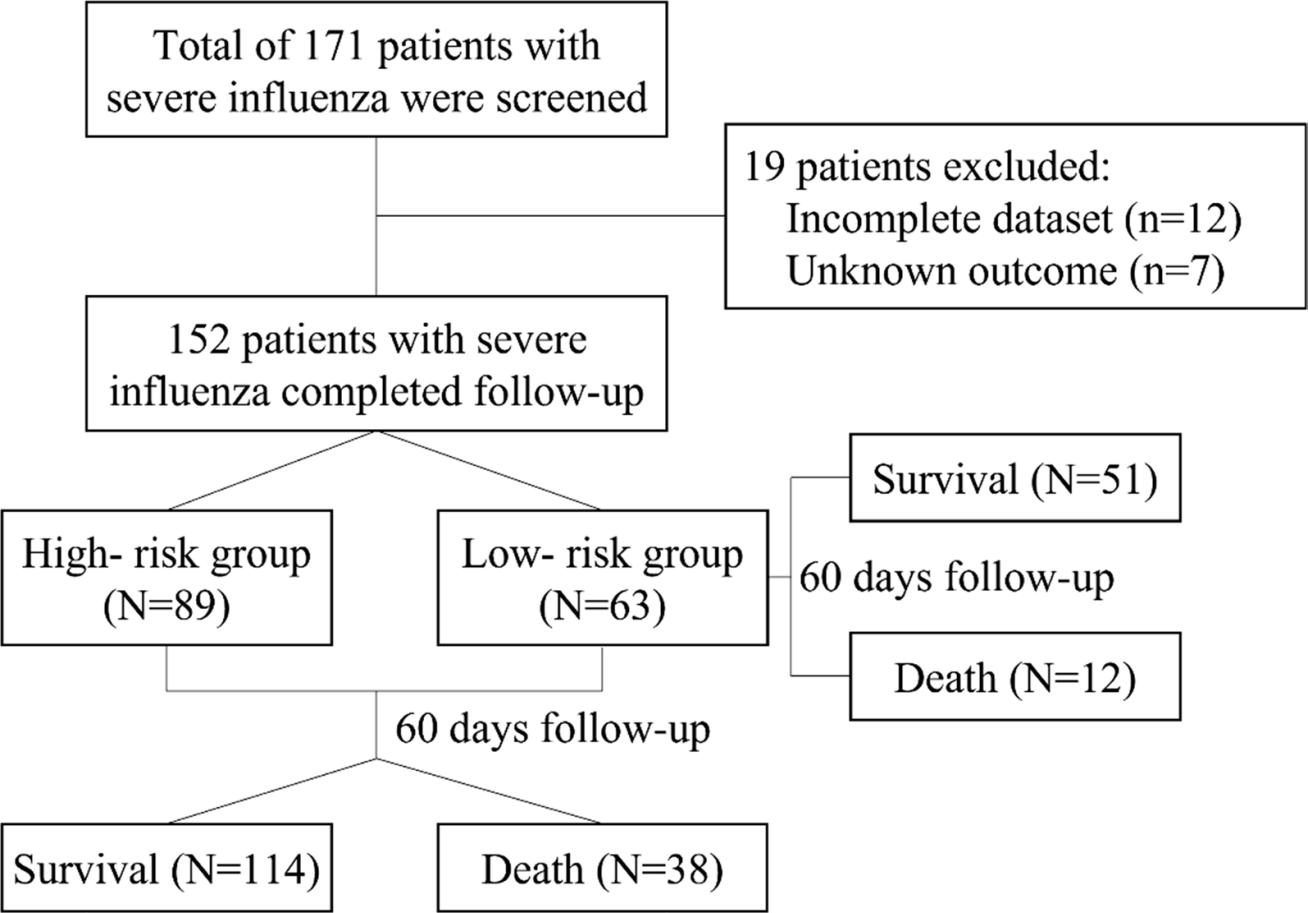
Flow chart of screening the study population

Data for 168 patients were included in the analysis. Of these, 16 patients were excluded due to incomplete clinical data or unclear prognoses, leaving a total of 152 patients in the study. The flowchart was shown as Fig. 1. The group having high-risk factors for severe influenza had 89 patients (Table 1), and the low-risk group included 63 patients who had no known high risk factors. All enrolled patients were diagnosed with pneumonia. 81 patients met the diagnostic criteria for severe pneumonia at the time of enrollment. Elderly, endocrine and cardiovascular diseases were the main risk factors for patients included in this study.

**Table 1:** Potential risk factors of patients in the high-risk group

Potential risk factor	No. of patients
Age ≥ 65	39/89
Chronic respiratory disease	21/89
Cardiovascular disease (hypertension excepted)	16/89
Chronic renal failure	5/89
Chronic hepatopathy	4/89
Hemopathy	5/89
Neurological and neuromuscular disorders	12/89
Endocrine disorders	23/89
Immunosuppression	14/89
Obesity	13/89
Pregnant and postpartum women	8/89

The demographic characteristics, disease conditions, and prognoses of patients in the two groups are shown in Table 2. There were more female patients in the high-risk group compared to the low-risk group. There was no statistical difference between the two groups in terms of time from onset of symptoms to hospital admission, length of stay, mechanical ventilation, use of vasoactive drugs, and utilization rate of CRRT. Similarly, there was no significant difference between the two groups in terms of ICU admission rate as well as the proportion of moderate-to-severe ARDS and mortality. There was no significant difference in the proportion of severe pneumonia between the two groups. But patients in the low-risk group showed a higher proportion of severe pneumonia, which caused our concern. For both groups the median PSI, PIRO-CAP and CURB-65 score was 3.0 points, 3.0 points and 1.0 point, respectively, whereas the PSI and CURB-65 score was higher for the high-risk group than the low-risk group.

**Table 2:** Demographic information, disease status and prognosis of patients in high- and low-risk groups

	Total population (n = 152)	High-risk group (n = 89)	Low-risk group (n = 63)	*p* value
Age (yrs)	52.9 ± 15.7	57.0 ± 16.9	47.1 ± 11.7	< 0.001
Gender (male/female ratio)	1:0.42	1:0.65	1:0.19	0.002
Time from onset of symptoms to hospital admission (days)	7.0 (5.0, 9.0)	7.0 (5.0, 9.5)	7.0 (4.0, 9.0)	0.800
Length of hospital stay (days)	12.0 (7.0, 23.0)	13.0 (8.0, 21.5)	11.0 (6.0, 26.0)	0.718
Ventilatory strategies (n, (%))				
Non-invasive ventilation	38 (25.00%)	21 (23.60%)	17 (26.98%)	0.636
Invasive mechanical ventilation	69 (45.39%)	39 (43.82%)	30 (47.62%)	0.644
ECMO	30 (19.74%)	14 (15.73%)	16 (25.40%)	0.142
Use of vasoactive drugs* (n, (%))	49 (32.24%)	29 (32.58%)	20 (31.75%)	0.914
CRRT (n, (%))	22 (14.47%)	12 (13.48%)	10 (15.87%)	0.647
ICU admission (n, (%))	83 (54.61%)	45 (50.56%)	38 (60.32%)	0.236
Moderate to severe ARDS** (n, (%))	79 (51.97%)	44 (49.44%)	35 (55.56%)	0.459
Severe pneumonia (n, (%))	91 (59.87%)	50 (56.18%)	41 (65.08%)	0.261
60-day death (n, (%))	38 (25.00%)	26 (29.21%)	12 (19.05%)	0.155
PSI (at admission)	3.0 (2.0, 4.0)	3.0 (2.0, 4.0)	2.0 (1.0, 3.0)	0.004
PIRO-CAP (at admission)	3.0 (3.0, 4.0)	3.0 (3.0, 4.0)	3.0 (3.0, 3.0)	0.084
CURB-65 (at admission)	1.0 (0, 2.0)	1.0 (1.0, 2.0)	1.0 (0, 2.0)	0.025

*Vasoactive drugs including dopamine and noradrenaline

**Moderate to severe ARDS refers to Berlin standard: PEEP ≥ 5cmH_2_O, PaO_2_/FIO_2_ ≤ 200 mmHg

All patients included in this study, as well as patients in the low-risk group were stratified according to PSI, CURB-65 and PIRO-CAP scores. The findings and actual 30-day mortality rate, as well as the predictive mortality rate [13–15] are shown in Table 3. For the PSI assessment, 72.4% of patients had the stage of I–III, but the mortality rate for this subgroup accounted for 48.6% of deaths among all patients. In low-risk group, patients classified in the stage I–III accounted for 84.1% of all low-risk patients, but the mortality rate accounted for 66.7% of deaths of all low-risk patients. According to the CURB-65 assessment, 87.5% of patients had scores of 0–2 points, but the mortality rate for this subgroup accounted for 67.6% of deaths among all patients. Such a finding was more prominent for low-risk patients in those patients having scores of 0–2 points accounted for 88.9% of all low-risk patients, but the mortality rate accounted for 75% of deaths of all low-risk patients. According to the PIRO-CAP assessment, 73.7% of patients had low- or moderate risk (scores of 0–3 points), and this group represented 43.2% of deaths among all patients. In the low-risk group, 84.1% were classified as having low- and mild- risk, and the mortality rate accounted for 66.7% of deaths of all low-risk patients. Therefore, For influenza pneumonia, commonly used assessment tools did not show the same predictive power as for other types of CAP.

**Table 3:** Predicted and actual 30-day mortality rates among hospitalized patients with influenza pneumonia stratified by PSI, CURB-65 and PIRO-CAP

	Total population (n = 152)			Low-risk group (n = 63)			Predicted mortality (%)
	Patients (No.)	Deaths (No.)	Actual mortality (%)	Patients (No.)	Deaths (No.)	Actual mortality (%)	
*PSI*							
I	36	2	5.6	19	1	5.3	0.1
II	36	7	19.4	18	4	22.2	0.6
III	38	9	23.7	16	3	18.8	0.9
IV	33	14	42.4	7	2	28.6	9.3
V	10	5	50.0	3	2	66.7	27.0
*CURB-65*							
0	44	2	4.5	26	1	3.8	0.7
1	50	9	18.0	17	3	17.6	2.1
2	39	14	35.9	13	5	38.5	9.2
3	13	7	53.8	5	2	40.0	14.5
4	5	4	80.0	2	1	50.0	40.0
5	1	1	100.0	0	0	0	57.0
*PIRO-CAP*							
Low risk (0–2)	32	2	6.3	14	0	0	3.6
Mild risk (3)	80	14	17.5	39	8	20.5	13.0
High risk (4)	30	14	46.7	9	3	33.3	43.0
Very high (≥ 5)	10	7	70.0	1	1	100.0	76.3

### Risk factors of influenza A-related pneumonia mortality in patients with different risks

The observation time for assessment of mortality in this study was 60 d, and the endpoint was hospitalization for 60 d or death. In the high-risk group, age, BMI, lymphocyte count, albumin, prealbumin, total cholesterol, high density lipoprotein cholesterol (HDL-C), low density lipoprotein cholesterol (LDL-C), lactic dehydrogenase (LDH), hydroxybutyrate dehydrogenase (HBDH), direct bilirubin, prothrombin activity (PTA), D-dimer, serum ferritin, C-reactive protein, CD3^+^ T cells, CD4^+^ T cells, CD8^+^ T cells, procalcitonin (PCT), pulmonary bacterial infection, and bacteremia were significantly different between the survival and mortality subgroups (Additional file 1). The variables with statistical significances were included in a Logistic regression equation, which showed that D- dimer (OR = 1.074, 95% CI 1.005–1.149, *p* = 0.036) was independent risk factor of influenza A-related pneumonia mortality of patients in the high-risk group. And CD4^+^T cells count (OR = 0.994, 95%CI 0.988–0.999, *p* = 0.023) was protective factor of patients in the high-risk group.

In the low-risk group, platelet, total protein, albumin, total cholesterol, LDL-C, aspartate aminotransferase (AST), HBDH, alkaline phosphatase (ALP), gamma-glutamyl transpeptidase (GGT), total bilirubin, direct bilirubin, blood urea nitrogen (BUN), D-dimer, serum ferritin, complement C3, and PCT significantly differed between the survival and mortality subgroups (Additional file 1). When variables with statistical significances were included in a Logistic regression equation, D-dimer (OR = 1.101, 95%CI 1.016–1.193, *p* = 0.019) and direct bilirubin (OR = 1.235, 95%CI 1.055–1.446, *p* = 0.009) were found to be independent risk factors of influenza A-related pneumonia mortality of patients in the low-risk group.

Of the above-mentioned variables that had significant differences between the survival and mortality groups, age, AST, HBDH, GGT, and complement C3 were also significantly different between the high- and low- risk groups. Albumin, total cholesterol, LDL-C, direct bilirubin, D-dimer, serum ferritin, and PCT were significantly different between the survival versus mortality subgroups for both high- and low-risk patients. Variables with statistical significances included in a Logistic regression equation again showed that D-dimer (OR = 1.052, 95%CI 1.001–1.106, *p* = 0.045) and direct bilirubin (OR = 1.143, 95%CI 1.049–1.246, *p* = 0.002) was an independent risk factor of influenza A-related pneumonia mortality of patients.

### Predictive value of severity assessment tools with D-dimer and ferritin for prognoses of patients with severe influenza A-related pneumonia

PSI, CURB-65 and PIRO-CAP assessment was performed for all patients with influenza A-related pneumonia included in this study (Table 4). The endpoint of this part was hospitalization for 30 d or death. For PSI assessment, the score for patients was 2.0 (1.0, 3.0) and 4.0 (2.5, 4.0) in the survival group and mortality group, respectively, and the difference was statistically significant (*p* < 0.001). The analysis of the PSI ROC curve that differentiated the survival group and the mortality group of patients with influenza A-related pneumonia showed that the AUC was 0.690 (95%CI 0.581–0.799, *p* = 0.002).

**Table 4:** AUC of different detection combinations

Prediction system	All patients			Low- risk group			High- risk group		
	AUC	95%CI	*p* value*	AUC	95%CI	*p* value*	AUC	95%CI	*p* value*
PSI	0.690	0.581–0.799		0.661	0.507–9.794		0.696	0.569–0.804	
PSI + D-dimer	0.793	0.702–0.884	0.013	0.782	0.635–0.890	0.109	0.810	0.694–0.897	0.061
PSI + D-dimer + Ferritin	0.851	0.780–0.922	0.002	0.839	0.701–0.931	0.009	0.849	0.739–0.926	0.021
CURB-65	0.759	0.664–0.854		0.736	0.586–0.855		0.773	0.652–0.868	
CURB-65 + D-dimer	0.804	0.720–0.888	0.184	0.796	0.652–0.900	0.284	0.815	0.699–0.900	0.081
CURB-65 + D-dimer + Ferritin	0.840	0.775–0.925	0.031	0.808	0.665–0.909	0.176	0.852	0.743–0.928	0.090
PIRO-CAP	0.699	0.588–0.811		0.638	0.483–0.774		0.732	0.608–0.835	
PIRO-CAP + D-dimer	0.781	0.685–0.876	0.023	0.792	0.647–0.898	0.040	0.776	0.655–0.870	0.209
PIRO-CAP + D-dimer + Ferritin	0.829	0.748–0.911	0.003	0.813	0.671–0.913	0.031	0.832	0.719–0.913	0.076

*Compared with original disease assessment tools (PSI, CURB-65 and PIRO-CAP)

For CURB-65 assessment, the score for patients was 1.0 (0, 2.0) and 2.0 (1.0, 3.0) in the survival group and mortality group, respectively, and the difference was statistically significant (*p* < 0.001). The analysis of the CURB-65 ROC curve that differentiated the survival group and the mortality group of patients with influenza A-related pneumonia showed that the AUC was 0.759 (95%CI 0.664–0.854, *p* < 0.001). In the PIRO-CAP assessment, the score was 3.0 (2.0, 3.0) and 4.0 (3.0, 4.0) in the survival group and mortality group, respectively, and the difference was statistically significant (P < 0.001). Analysis of the PIRO-CAP ROC curve that differentiated the survival group and the mortality group of patients with influenza A-related pneumonia showed that the AUC was0.699 (95%CI 0.588–0.811, *p* < 0.001).

As D-dimer was an independent risk factor of influenza A-related pneumonia, the prediction value of these tools in combination with D-dimer for mortality was assessed. The findings showed that the AUC of ROC of PSI + D-dimer was 0.793 (95%CI 0.702–0.884, *p* < 0.001). Compared with PSI, PSI + D-dimer had higher predictive accuracy than PSI (*p* = 0.013). The AUC of ROC of CURB-65 + D-dimer was 0.804 (95%CI 0.720–0.888, *p* < 0.001). Compared with CURB-65, CURB-65 + D-dimer improved the prediction accuracy, but the difference was not statistically (*p* = 0.184). And the AUC of ROC of PIRO-CAP + D-dimer was 0.781 (95%CI 0.685–0.876, *p* < 0.001). Compared with PIRO-CAP, PIRO-CAP + D-dimer improved the prediction accuracy, and the difference was statistically significant (*p* = 0.023).

Previous studies demonstrated that serum ferritin has predictive potential for poor prognoses including respiratory failure, ICU stay, and death [16]. This study also demonstrated that ferritin significantly differed between the survival group and mortality group, and thus the prediction value of the three assessment tools in combination with ferritin and D-dimer for mortality was assessed. The AUC of ROC of PSI + ferritin + D-dimer was 0.851 (95%CI 0.780–0.922, *p* < 0.001). The AUC of ROC of CURB-65 + ferritin + D-dimer was 0.840 (95%CI 0.763–0.916, *p* < 0.001), The AUC of ROC of PIRO-CAP + ferritin + D-dimer was 0.829 (95%CI 0.748–0.911, *p* < 0.001). When these assessment tools combined with ferritin and D- dimer, the predictive values were better than the tools alone. The differences were statistically significant, the *p*-values were 0.002, 0.031 and 0.003, respectively.

In low-risk group, the AUCs of the three disease assessment tools were smaller than the AUCs in high- risk group. After combining D-dimer and ferritin, although the AUCs of the low- risk group were still smaller than that of the high-risk group, the improvements of the AUCs of PSI and PIRO-CAP were better than that of the high-risk group, which also made them more beneficial in the combined detection process.

## Discussion

In this study, we addressed the possibility that unknown potential risk factors may increase the likelihood that patients in a low-risk population will progress to severe disease, and have the same mechanical ventilation rate, ICU hospitalization rate and fatality rate as high-risk patients. In this study, we found that D-Dimer was an independent risk factor for death of hospitalized patients with severe influenza A, and the combination of D-Dimer and ferritin could reduce the underestimation of the fatality rate of existing CAP disease severity scoring systems for influenza A pneumonia, thus improving its predictive value. These findings contribute to enhanced understanding of factors associated with severe influenza and improve the ability of clinicians to predict an adverse prognosis of patients with severe influenza A.

For high-risk patients with severe influenza, diagnosis and treatment approaches have been standardized in guidelines that were developed by various groups, such as the Infectious Diseases Society of America, and in this study these approaches defined which patients were classified into high-risk group and low-risk groups. Approximately 60% of patients in the study had high-risk factors for progression to severe influenza. Advanced age, chronic respiratory diseases (mainly COPD and interstitial lung disease), and endocrine disease (mainly diabetes) were the major high-risk factors. The existence of such high-risk factors highlights the need for clinicians to monitor members of this high-risk population closely.

However, of those patients in the study who did progress to severe influenza, over 40% had no such high-risk factors. The percentage of patients with no known risk factors who required mechanical ventilation as well as the survival rate after CRRT treatment, rate of use of vasoactive drugs, ICU stay rate, percentage who progressed to moderate or severe ARDS, and even the mortality rate did not significant differ compared with high-risk patients. The CAP severity evaluation tools that are currently commonly used could not effectively and correctly assess poor prognoses of the patients, and could severely underestimate the mortality rate for low-risk patients. These findings emphasize the need to reassess patients with influenza A and determine the factors that were previously missed.

In contrast to high-risk patients, the low-risk population had more evident liver injuries and higher complement C3 levels. Previous studies suggested that the novel H1N1 influenza virus could cause direct liver injury effects [17]. These injuries were largely thought to be associated with disease severity, rather than an emerging of hepatotropic property of the virus [18]. The findings in this study showed that the severity of liver injuries was more prominent in the low-risk population, but the incidence was similar to that for high-risk patients. The most prominent theory was that liver injuries are immune-mediated and caused by molecular interactions between virus and hepatocyte antigens [19]. Individuals in low-risk populations had no other complications before the occurrence of severe influenza, which could make immune responses stronger and more readily detectable.

The complement system plays an important role in host immune responses during virus infection via regulatory effects of virus particles, aggregation of inflammatory cells, and lysis of infected cells [20]. In previous studies, mice with C3 knockout that were infected with other respiratory viruses such as SARS-CoV were shown to have reduced lung invasion of neutrophils and inflammatory monocytes, and reduced respiratory dysfunction [21]. Complement C3 levels in patients who did not survive were further decreased, and the differences in C3 between surviving patients and those patients who died were more apparent in the low-risk group. Such differences were more pronounced for C3 than for other immune factors and most inflammatory indicators, indicating that higher levels of C3 played a role in the pathogenic effects of influenza virus infection in low-risk patients. Further investigation of complement C3 levels or dynamic changes in this factor could provide additional support for the use of C3 as a predictor for poor prognoses of patients with severe influenza.

Patients with and without known risk factors for progression to severe influenza had significantly different nutritional status and inflammatory factors between patients who survived and those who did not. D-dimer was found to be an independent risk factor of influenza A-related pneumonia mortality. Earlier studies showed that elevations in D-dimer are associated with progression of novel H1N1 influenza [22, 23]. An association between thrombogenesis and viral pneumonia caused by influenza virus or novel coronavirus was also demonstrated, indicating that activation of the thrombosis system was associated with D-dimer elevation [24]. This theory could also explain the elevation in D-dimer levels seen for the mortality group in the present study, in that D-dimer was not only associated with disease progression, but could also be an important predictor of adverse prognoses.

Compared with patients with pneumonia caused by other pathogens, the age of disease onset for patients with viral pneumonia was relatively low, and the disease progression was rapid. Therefore, even during the initial phase of the disease, the predictive value of all known severity assessment tools for poor prognoses was reduced. In this study, the AUC of PSI, CURB-65 and PIRO-CAP for predicting 30-day mortality was < 0.8. In agreement with our findings, various previous studies demonstrated similar AUC values that ranged from 0.64 to 0.72 for CURB-65 and PIRO-CAP in predicting mortality of viral pneumonia [8, 9, 25]. Previous studies also indicated that the performance of severity assessment tools, including PSI, CURB-65 and PIRO-CAP, for predicting severity of viral pneumonia was reduced [8]. Thus, improved evaluation of disease conditions and predictive prognoses for patients with viral pneumonia is urgently needed for clinical practices.

The findings of this study demonstrated that combining D-dimer and ferritin values with PSI, CURB-65 and PIRO-CAP could effectively increase the AUC, and improve the ability to predict death within 30 days of developing severe influenza A pneumonia. Direct bilirubin, although considered an independent risk factor for 60-day death in these patients, did not improve the predictive power of these assessment tools. The addition of these factors could aid not only early identification of patients who have higher likelihood of developing severe disease, but also help guide allocation of treatment resources (e.g., ICU beds) and choice of more proactive treatments. In future studies, we will establish a predictive model combining D-dimer and ferritin to develop a more appropriate disease assessment tool for viral pneumonia. In particular, patients diagnosed with influenza pneumonia in low- risk group had no lower mortality than those diagnosed in high- risk group. Our study explored those new indicators to increase the predictive value of the original score for 30-day mortality, and these two indicators could benefit the low-risk group especially.

There were several limitations of this study. First, only 152 patients hospitalized with severe influenza at a single treatment center were enrolled. In addition, the authors were worked in a hospital which was a site for local diagnosis and treatment center for respiratory diseases. The overall disease conditions of the patients with severe influenza who were enrolled in the study were very complex, and thus a potential selection bias may exist. Additional detailed analyses that include data from other centers is needed to confirm and expand the findings described in this study and to help establish an ideal predictive model for early identification of patients who may progress to severe disease.

## Conclusions

In summary, the findings of this study demonstrated that patients with influenza A who develop severe disease may have unknown risk factors. Low-risk patients had similar rate of mechanical ventilation, ICU hospitalization rate, and mortality rate as the high-risk patients. Using the currently available CAP severity assessment system could lead to an underestimation of influenza A-related pneumonia mortality. D-dimer was an independent risk factor for influenza A-related pneumonia mortality of hospitalized patients. Including D-dimer and ferritin values could increase the predictive performance of PSI, CURB-65 and PIRO-CAP that are used to identify patients with influenza A-related pneumonia who have poor prognoses.

## Supplementary Information

**Additional file 1.** Partial demographic characteristics and laboratory results among different groups.

## Data Availability

The deidentified data and statistical analysis code that support the findings of this study are available on reasonable request from the corresponding author.

## Abbreviations

CAP: Community-acquired pneumonia
COPD: Chronic obstructive pulmonary disease
ARDS: Acute respiratory distress syndrome
ICU: Intensive care unit
PSI: Pneumonia severity index
IRB: Institutional review board
BMI: Body-mass index
CRRT: Continuous renal replacement therapy
RT-PCR: Reverse transcriptase polymerase chain reaction
OR: Odds ratio
CI: Confidence interval
ROC: Receiver operator characteristic curve
AUC: Area under the curve
HDL-C: High density lipoprotein cholesterol
LDL-C: Low density lipoprotein cholesterol
LDH: Lactic dehydrogenase
HBDH: Hydroxybutyrate dehydrogenase
PTA: Prothrombin activity
PCT: Procalcitonin
AST: Aspartate aminotransferase
ALP: Alkaline phosphatase
GGT: Gamma-glutamyl transpeptidase
BUN: Blood urea nitrogen
