# Supplementary material for: Additional risk factors improve mortality prediction for patients hospitalized with influenza pneumonia: a retrospective, single-center case–control study
Source: BMC Pulm Med. 2023 Jan 16;23:19. doi: 10.1186/s12890-022-02283-6 (PMC9841622; doi:10.1186/s12890-022-02283-6)
Supplement: Supplementary file 1 — Additional file 1. Partial demographic characteristics and laboratory results among different groups. [file 12890_2022_2283_MOESM1_ESM.docx]

| **Supplementary Table 1** Partial demographic characteristics and laboratory results among different groups | | | | | | | | | | | | |
| --- | --- | --- | --- | --- | --- | --- | --- | --- | --- | --- | --- | --- |
|  | High risk group | | | |  | Low risk group | | | |  | *p* value | |
|  | All  (n=89) | Survival Group  (n=63) | Death Group  (n=26) | *p* value |  | All  (n=63) | Survival Group  (n=51) | Death Group  (n=12) | *p* value |  |  |  |
| Age (yrs) | 57.0±16.9 | 54.2±17.6 | 63.8±12.8 | **0.006** |  | 47.1±11.7 | 46.5±11.2 | 49.7±13.7 | 0.407 |  | **<0.001** |  |
| Gender (Male/Female ratio) | 1:0.65 | 1:0.75 | 1:0.44 | 0.291 |  | 1:0.19 | 1:0.24 | 0 | 0.097 |  | **0.002** |  |
| Time from onset of symptoms to hospital admission (days) | 7.8±5.6 | 7.1±4.2 | 9.6±7.9 | 0.135 |  | 7.1±4.0 | 6.9±3.9 | 8.0±4.7 | 0.400 |  | 0.398 |  |
| Length of hospital stay (days) | 13.0 (8.0, 21.5) | 12.0 (8.0,22.0) | 17.00 (7.75, 21.25) | 0.850 |  | 11.0 (6.0, 26.0) | 12.0 (6.0, 26.0) | 7.5 (3.3, 28.3) | 0.192 |  | 0.718 |  |
| BMI (kg/m^2^) | 25.5±5.1 | 26.5±5.4 | 22.9±3.0 | **<0.001** |  | 24.8±3.3 | 24.9±3.5 | 24.8±2.3 | 0.917 |  | 0.392 |  |
| Smoker [n (%)] | 35 (39.3%) | 25 (39.7%) | 10 (38.5%) | 0.915 |  | 27 (42.9%) | 19 (37.3%) | 8 (66.7%) | 0.066 |  | 0.664 |  |
| Alcohol abuse [n (%)] | 16 (18.0%) | 12 (19.0%) | 4 (15.4%) | 0.684 |  | 10 (15.9%) | 7 (13.7%) | 3 (25.0%) | 0.340 |  | 0.735 |  |
| WBC (×10^9^/L) | 7.2±5.2 | 6.8±3.9 | 7.9±7.0 | 0.487 |  | 6.1±3.4 | 6.1±3.0 | 6.4±5.1 | 0.767 |  | 0.150 |  |
| Neutrophil (×10^9^/L) | 5.9±4.9 | 5.0±3.6 | 7.0±7.0 | 0.269 |  | 4.9±3.3 | 4.8±2.8 | 5.4±4.9 | 0.514 |  | 0.133 |  |
| Lymphocyte (×10^9^/L) | 0.9±0.6 | 1.0±0.7 | 0.7±0.6 | **0.024** |  | 0.9±0.6 | 0.9±0.6 | 0.6±0.4 | 0.058 |  | 0.754 |  |
| Hemoglobin (g/L) | 118.3±27.5 | 119.6±25.1 | 115.3±33.1 | 0.510 |  | 136.8±18.0 | 138.6±17.4 | 129.3±19.4 | 0.111 |  | **<0.001** |  |
| Hematokrit (%) | 34.9±6.8 | 34.8±6.9 | 35.1±6.4 | 0.843 |  | 39.9±5.2 | 40.3±5.2 | 38.0±4.8 | 0.150 |  | **<0.001** |  |
| Platelet (×109/L) | 160.7±82.0 | 166.8±85.7 | 145.9±71.6 | 0.276 |  | 177.9±105.5 | 197.6±105.3 | 94.0±53.3 | **0.002** |  | 0.260 |  |
| Total protein (g/L) | 60.0±10.9 | 60.9±11.1 | 57.9±10.4 | 0.237 |  | 59.1±8.9 | 60.9±8.1 | 51.8±8.4 | **0.001** |  | 0.603 |  |
| Albumin (g/L) | 32.5±6.0 | 33.5±6.1 | 30.1±5.0 | **0.014** |  | 33.8±5.7 | 35.1±5.1 | 28.5±5.2 | **<0.001** |  | 0.173 |  |
| Globulin (g/L) | 27.4±7.9 | 27.4±8.3 | 27.4±7.1 | 0.970 |  | 25.3±4.6 | 25.8±4.4 | 23.3±4.9 | 0.097 |  | 0.041 |  |
| Total cholesterol (mmol/L) | 3.6±1.2 | 3.8±1.2 | 3.1±1.1 | **0.010** |  | 3.3±0.8 | 3.4±0.8 | 2.9±0.9 | **0.035** |  | 0.081 |  |
| High-density lipoprotein (mmol/L) | 0.9±0.4 | 0.9±0.4 | 0.7±0.4 | **0.004** |  | 0.8±0.3 | 0.8±0.3 | 0.7±0.4 | 0.136 |  | 0.133 |  |
| Low-density lipoprotein (mmol/L) | 1.8±0.9 | 2.0±0.9 | 1.4±0.7 | **0.001** |  | 1.7±0.7 | 1.8±0.7 | 1.3±0.6 | **0.021** |  | 0.330 |  |
| Triglycerides (mmol/L) | 1.8±1.1 | 1.8±1.2 | 1.9±0.9 | 0.590 |  | 2.1±1.6 | 2.0±1.6 | 2.3±1.4 | 0.640 |  | 0.209 |  |
| AST (U/L) | 39.0 (27.5, 63.0) | 38.0 (27.0, 58.0) | 46.5 (29.8, 71.8) | 0.277 |  | 59.0 (37.0, 132.0) | 50.0 (35.0, 106.0) | 93.0 (70.8, 157.5) | **0.022** |  | **<0.001** |  |
| ALT (U/L) | 25.0 (16.5, 39.0) | 25.0 (16.0, 52.0) | 22.5 (16.5, 32.0) | 0.396 |  | 42.0 (25.0, 76.0) | 49.0 (24.0, 69.0) | 52.5 (32.0, 129.8) | 0.169 |  | **<0.001** |  |
| Creatine kinase (umol/L) | 143.0 (51.0, 349.5) | 142.0 (54.0, 375.0) | 146.5 (48.0, 322.0) | 0.573 |  | 162.0 (85.0, 432.0) | 155.0 (74.0, 403.0) | 182.5 (95.0, 757.3) | 0.386 |  | 0.130 |  |
| LDH (U/L) | 468.9±350.7 | 395.7±268.6 | 646.2±455.9 | **0.013** |  | 687.4±557.6 | 639.8±590.2 | 889.6±337.9 | 0.164 |  | **0.007** |  |
| HBDH (U/L) | 375.2±249.2 | 318.0±195.9 | 513.7±308.5 | **0.005** |  | 496.4±272.5 | 457.6±267.1 | 661.2±240.0 | **0.019** |  | **0.005** |  |
| ALP (U/L) | 85.0±44.0 | 78.6±39.2 | 100.6±51.5 | 0.058 |  | 95.1±73.9 | 73.8±37.8 | 185.3±115.5 | **0.007** |  | 0.339 |  |
| GGT (U/L) | 32.0 (18.5, 64.0) | 32.0 (21.0, 59.0) | 32.0 (16.8, 94.0) | 0.989 |  | 65.0 (23.0, 121.0) | 53.0 (19.0, 115.0) | 177.0 (93.0, 473.0) | **0.006** |  | **0.010** |  |
| Total Bilirubin (umol/L) | 10.0 (6.8, 15.6) | 12.3±8.4 | 14.2±9.2 | 0.344 |  | 12.2 (7.3, 19.2) | 10.4 (7.1, 15.3) | 21.4 (16.5, 42.7) | **0.001** |  | 0.182 |  |
| Direct Bilirubin (umol/L) | 4.5 (2.6, 7.1) | 3.5 (2.5, 6.6) | 6.2 (3.9, 7.6) | **0.032** |  | 5.1 (3.4, 10.4) | 4.8 (2.9, 7.8) | 13.2 (9.0, 25.5) | **0.001** |  | 0.058 |  |
| Blood urea nitrogen (mmol/L) | 8.10±5.84 | 7.48±6.24 | 9.59±4.48 | 0.121 |  | 6.6±4.4 | 5.8±3.7 | 10.2±5.5 | **0.020** |  | 0.098 |  |
| Creatinine (umol/L) | 97.37±87.23 | 97.84±91.40 | 96.23±77.86 | 0.937 |  | 92.5±71.9 | 82.8±50.4 | 133.8±123.8 | 0.187 |  | 0.715 |  |
| Uric Acid (umol/L) | 246.5±116.5 | 255.9±119.4 | 223.7±108.0 | 0.238 |  | 241.0±125.7 | 242.4±118.5 | 235.0±158.7 | 0.856 |  | 0.782 |  |
| PT (s) | 14.1±8.4 | 13.0±3.1 | 16.8±14.5 | 0.190 |  | 12.7±2.7 | 12.5±2.5 | 13.8±3.9 | 0.137 |  | 0.215 |  |
| PA (%) | 81.3±17.8 | 84.2±15.0 | 74.4±22.0 | **0.018** |  | 83.4±14.0 | 84.7±13.1 | 78.0±16.6 | 0.139 |  | 0.449 |  |
| APTT (s) | 30.7 (25.6, 35.4) | 31.0±8.4 | 36.8±13.5 | 0.051 |  | 27.4 (25.0, 36.6) | 27.3 (24.9, 35.9) | 30.0 (25.0, 54.6) | 0.224 |  | 0.355 |  |
| Fibrinogen (mg/dl) | 395.1±126.1 | 404.2±115.9 | 373.4±140.1 | 0.299 |  | 420.1±144.0 | 429.6±146.5 | 379.7±131.0 | 0.283 |  | 0.259 |  |
| D-dimer (mg/L FEU) | 5.9±9.3 | 1.6 (0.9, 3.9) | 3.3 (1.7, 10.7) | **0.003** |  | 7.4±9.9 | 5.7±8.8 | 13.8±11.5 | **0.010** |  | 0.353 |  |
| Serum ferritin (ng/ml) | 860.6 (362.3, 1886.4) | 603.6 (323.2, 1331.7) | 1439.4 (657.3, 3835.5) | **0.003** |  | 1234.5 (643.2, 2821.1) | 1071.9 (467.7, 1917.8) | 2891.8 (1745.2, 3424.1) | **0.005** |  | 0.062 |  |
| ESR (mm/h) | 26.4±22.6 | 20.0 (15.5, 28.8) | 18.0 (6.5, 43.8) | 0.687 |  | 21.4±14.8 | 22.6±15.4 | 16.8±12.0 | 0.228 |  | 0.117 |  |
| CRP (mg/dl) | 11.1±9.4 | 7.4 (2.5, 13.4) | 13.9 (8.6, 18.4) | **0.002** |  | 11.3±10.4 | 10.1±10.3 | 17.2±9.1 | 0.063 |  | 0.895 |  |
| IgG (mg/dl) | 954.0±786.5 | 968.7±895.9 | 924.7±516.0 | 0.818 |  | 926.6±283.0 | 946.8±292.4 | 853.8±243.5 | 0.319 |  | 0.777 |  |
| IgA (mg/dl) | 188.4±137.1 | 187.3±129.9 | 190.5±153.0 | 0.922 |  | 188.1±85.7 | 194.9±69.6 | 163.9±129.3 | 0.272 |  | 0.988 |  |
| IgM (mg/dl) | 67.4 (37.1, 97.4) | 67.4 (36.9, 97.7) | 65.4 (37.0, 95.7) | 0.899 |  | 60.7 (46.0, 93.7) | 67.7±29.9 | 105.1±67.2 | 0.085 |  | 0.739 |  |
| Complement 3 (mg/dl) | 67.7±31.5 | 69.2±31.8 | 64.7±31.3 | 0.554 |  | 78.1±25.4 | 82.3±25.1 | 62.7±20.8 | **0.016** |  | **0.045** |  |
| Complement 4 (mg/dl) | 22.5±12.6 | 22.2±11.7 | 23.2±14.5 | 0.757 |  | 24.6±8.6 | 25.7±8.7 | 20.5±7.0 | 0.060 |  | 0.274 |  |
| CD3^+^T cell (/ul) | 367.0 (203.5, 677.5) | 480.0 (251.0, 869.0) | 230.0 (112.8, 230.0) | **0.001** |  | 357.0 (238.5, 473.8) | 363.0 (240.5, 542.8) | 344.5 (198.5, 465.5) | 0.607 |  | 0.933 |  |
| CD4^+^T cell (/ul) | 224.0 (113.5, 433.5) | 301.0 (165.0, 569.0) | 135.0 (35.0, 216.8) | **0.001** |  | 230.0 (154.0, 327.5) | 212.0 (150.0, 387.0) | 247.5 (149.0, 295.5) | 0.971 |  | 0.948 |  |
| CD8^+^T cell (/ul) | 132.0 (68.5, 221.5) | 152.0 (82.0, 262.0) | 103.0 (41.3, 143.5) | **0.007** |  | 119.0 (80.0, 199.0) | 119.0 (81.0, 188.0) | 108.50 (72.0, 214.5) | 0.687 |  | 0.900 |  |
| PCT (ng/ml) | 0.4 (0.1, 1.7) | 0.3 (0.1, 1.1) | 1.1 (0.5, 5.5) | **0.001** |  | 0.5 (0.1, 3.8) | 0.3 (0.1, 2.5) | 2.5 (0.5, 7.0) | **0.012** |  | 0.920 |  |
| Co-infection with bacteria [n (%)] | 25 (28.1%) | 11 (17.5%) | 14 (53.8%) | **0.002** |  | 25 (39.7%) | 18 (35.3%) | 7 (58.3%) | 0.145 |  | 0.116 |  |
| Co-infection with fungi [n (%)] | 38 (42.7%) | 22 (34.9%) | 16 (61.5%) | **0.022** |  | 28 (41.3%) | 24 (47.1%) | 4 (33.3%) | 0.393 |  | 0.831 |  |
| Bacterimia [n (%)] | 10 (11.2%) | 2 (3.2%) | 8 (30.8%) | **<0.001** |  | 6 (9.5%) | 4 (7.8%) | 2 (16.7%) | 0.353 |  | 0.736 |  |
